# Damage-associated molecular pattern recognition is required for induction of retinal neuroprotective pathways in a sex-dependent manner

**DOI:** 10.1038/s41598-018-27479-x

**Published:** 2018-06-14

**Authors:** Marcus J. Hooper, Jiangang Wang, Robert Browning, John D. Ash

**Affiliations:** Department of Ophthalmology, University of Florida, Gainesville, FL 32610 USA

## Abstract

Retinal degeneration is a common cause of irreversible blindness and is caused by the death of retinal light-sensitive neurons called photoreceptors. At the onset of degeneration, stressed photoreceptors cause retinal glial cells to secrete neuroprotective factors that slow the pace of degeneration. Leukemia inhibitory factor (LIF) is one such factor that is required for endogenous neuroprotection. Photoreceptors are known to release signals of cellular stress, called damage-associated molecular patterns (DAMPs) early in degeneration, and we hypothesized that receptors for DAMPs or pattern recognition receptors (PRRs) play a key role in the induction of LIF and neuroprotective stress responses in retinal glial cells. Toll-like receptor 2 (TLR2) is a well-established DAMP receptor. In our experiments, activation of TLR2 protected both male and female mice from light damage, while the loss of TLR2 in female mice did not impact photoreceptor survival. In contrast, induction of protective stress responses, microglial phenotype and photoreceptor survival were strongly impacted in male TLR2^−/−^ mice. Lastly, using publicly available gene expression data, we show that TLR2 is expressed highly in resting microglia prior to injury, but is also induced in Müller cells in inherited retinal degeneration.

## Introduction

Retinal degenerative disorders are the largest cause of irreversible blindness in industrialized nations. Despite the presence of a deleterious mutation, retinal degeneration often proceeds slowly, and useful vision remains in humans for many years. Studies from our laboratory and others have shown that stress-inducible endogenous neuroprotective pathways are critical for reducing the rate of degeneration during disease progression^1–4^.

LIF is a well-established protective factor, and in the retina, LIF is critical for preservation of photoreceptors in light damage (LD) and inherited retinal degeneration^1,2,4,5^. Neuroprotective pathways in the retina are diverse, but often converge on the same downstream targets. One of these is STAT3, a transcription factor downstream of LIF^1–4,6,7^. In stress, STAT3 activation occurs in retinal neurons and glial cells^3,5^. Neuroprotective pathways, including those activated by LIF, are stress-inducible. It has been suggested that stress responses have evolved to protect photoreceptors from infection, retinal detachment, trauma and extended exposure to sunlight^8^. Consistent with this hypothesis, ligands of pattern recognition receptors (PRRs) have been shown to be induced by injury^9,10^. These ligands are derived from stressed or damaged photoreceptor cells, and are therefore referred to as damage-associated molecular patterns (DAMPs)^9–12^. These DAMPs include oxidized lipids and proteins, carboxyethylpyrrole-modified proteins, oxidized LDL, and 4-hydroxynonenal^9–13^. Many of these DAMPs, including carboxyethylpyrrole, a well-studied product of DHA oxidation have been identified as ligands to toll-like receptor 2 (TLR2), making TLR2 a good candidate for inducing protective stress responses in the retina^9,11,14–18^. In further support of TLR2 playing a role in protective stress responses, studies have shown that TLR2 activation induces neurotrophic factors and IL-6 family cytokines^19–23^. Additionally, TLR2 expression is increased in stressed retinas^24,25^. Lastly, TLR2 has been shown to influence microglial migration and to be protective in injury models in the CNS^26,27^. All of these results suggest that TLR2 may be important for the retinal response to stress and may play a role in LIF induction.

## Results

### Retinal structure and function in TLR2^−/−^ mice

**Figure 1:**
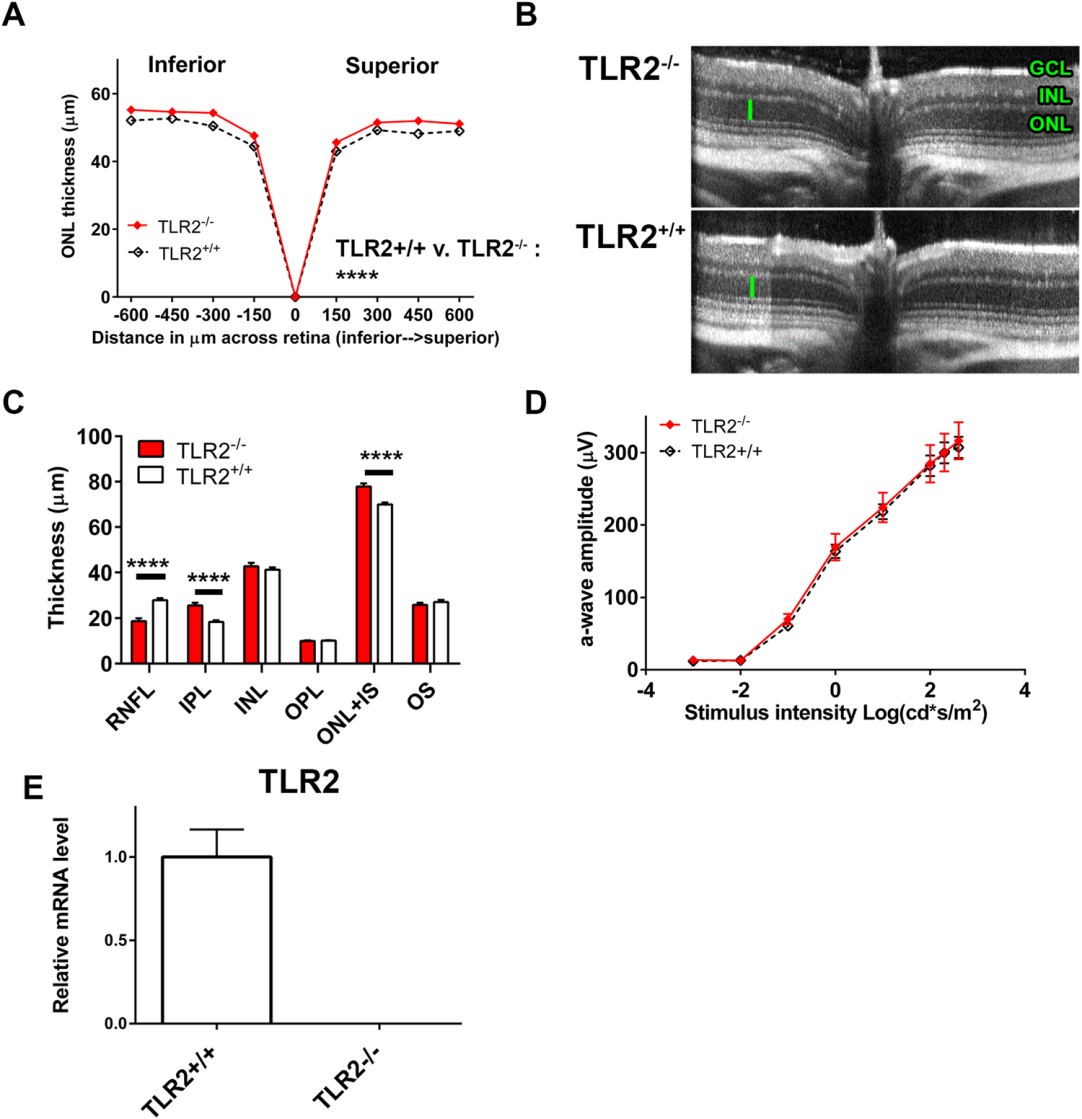
Retinal structure and function in TLR2^−/−^ mice. (**A**) ONL thickness of 6 to 10-week-old TLR2^−/−^ and TLR2^+/+^ mice is plotted versus distance from the optic nerve. (**B**) Representative OCT b-scans of TLR2^−/−^ and TLR2^+/+^ mice. (**C**) Thickness of retinal layers was measured using auto-segmentation in Diver software. (**D**) Scotopic ERG a-wave amplitudes of 4–8 week old TLR2^−/−^ and TLR2^+/+^ mice. (**E**) TLR2 mRNA expression to confirm TLR2 knockout in TLR2^−/−^ mice. Statistics: Two-way ANOVA (multiple comparisons were done for C). (**A**–**D**) n = 15–25 from three independent experiments. (**E**) n = 6–8 total from two independent experiments). The “No LD” group is made up of an average of all mice (all genders and genotypes) in the absence of light damage.

To investigate the role of TLR2 in LIF induction and endogenous neuroprotection, we crossed TLR2^−/−^ mice for more than 6 generations onto the Balb/cJ background. We measured photoreceptor layer (outer nuclear layer, ONL) thickness by Spectral-Domain Optical Coherence Tomography (OCT), and found that TLR2^−/−^ mice had a slight, but statistically significant increase in ONL thickness (Fig. 1A). Retinal layer auto-segmentation analysis confirmed the slight increase in the ONL-IS (ONL + photoreceptor inner segment). TLR2^−/−^ mice also displayed a slight increase in the inner plexiform layer (IPL) thickness and a slight decrease in the retinal nerve fiber layer (RNFL) thickness (Fig. 1C). The significance of these baseline changes is unknown, but is consistent with observations that TLR2^−/−^ mice have reduced axonal lengths in the enteric nervous system and reduced optic nerve axon regeneration following injury^19,28^. TLR2^−/−^ mice had normal full-field scotopic ERG responses (Fig. 1D) relative TLR2^+/+^ controls, suggesting that retinal function was not affected by these small changes in layer thicknesses. There were no differences in retinal structure, layer thicknesses, or ERG a-wave responses between sexes from either genotype, we did however observe a small, but statistically significant difference in b-wave amplitudes when comparing TLR2^−/−^ males and females (Fig. S1). To confirm deletion in TLR2^−/−^ mice, we measured TLR2 expression using qRT PCR. TLR2^−/−^ mice did not express TLR2 at the mRNA level (Fig. 1E). We conclude that under normal conditions TLR2^−/−^ mouse retinas have slight changes in retinal structure when compared with TLR2^+/+^ mice, but are very similar and seem to develop normally.

### TLR2 is necessary for stress responses and LIF induction in LD

**Figure 2:**
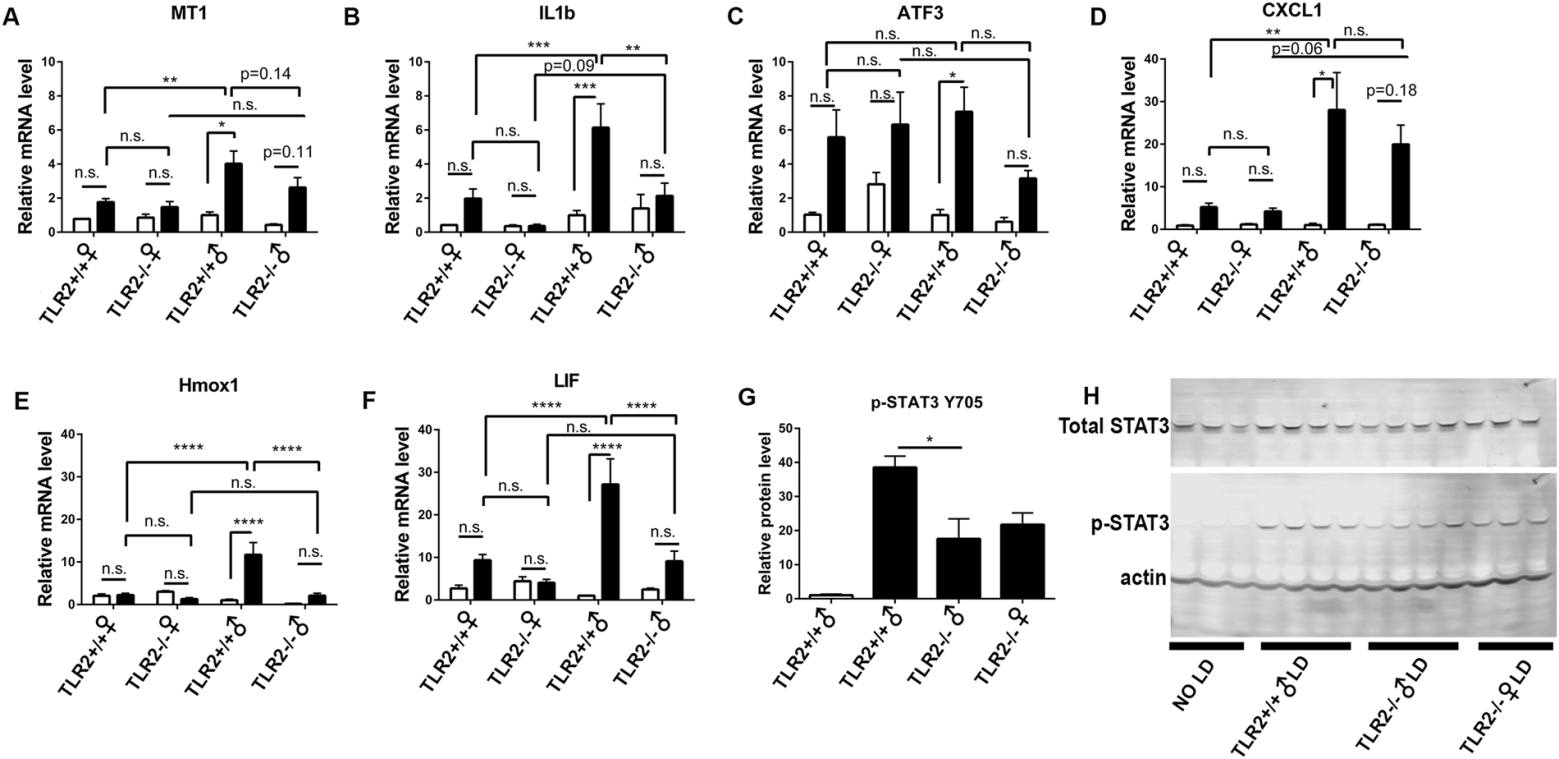
TLR2 is necessary for LIF induction and stress responses following light damage (LD). mRNA expression as measured by qRT-PCR following light damage is shown for (**A**) MT1, (**B**) IL-1b, (**C**) ATF3, (**D**) CXCL1, (**E**) Hmox1, (**F**) LIF. White bars: No LD controls, black bars: mice exposed to light damage. Y-axes are log_2_(fold change relative to TLR2^+/+^ males with no LD). Statistics: Two-way ANOVA with multiple comparisons. (**A**–**F**) n = 11–16 mice in sex-specific groups exposed to light damage. Mice with no light damage: n = 3–6. (**G**,**H**) Protein expression of phosphorylated STAT3 (pSTAT3) following LD in TLR2^−/−^ mice and controls. Statistics: unpaired, two-tailed t-test. (**G**,**H**) n = 3–4 mice, representative of 3 independent experiments. Western blots were cropped. Full, uncropped versions of these blots can be found in Figs S7 and S8.

Exposure to damaging light causes gene expression changes associated with injury, stress response, and neuroprotection in wild type mice^29^. Using quantitative real time PCR (qRT-PCR), we measured expression levels of genes in TLR2^+/+^ and TLR2^−/−^ mice, which are induced following light stress. These include MT1, IL1b, ATF3, CXCL1, Hmox1, LIF, and p-STAT3 (Fig. 2A–H). We found a surprising difference between male and female TLR2^+/+^ mice. As such, in our analysis, TLR2^+/+^ and TLR2^−/−^ mice were subdivided into sex-specific groups. Stress-inducible genes including MT1, IL1b, CXCL1, Hmox1, and LIF were more highly induced in male mice than in female mice. Importantly, when comparing genotypes, we found that induction of protective genes LIF, Hmox1 and pSTAT3 were reduced in TLR2^−/−^ mice relative to TLR2^+/+^ mice. These differences achieved statistical significance only when comparing TLR2^+/+^ and TLR2^−/−^ male mice (qPCR: Two-way ANOVA, Sidak’s post test, western blot: unpaired, two-tailed t-test). Interestingly, expression of microglia-specific genes IRF8, INOS, and CD206 were slightly altered when comparing untreated TLR2^−/−^ males with untreated TLR2^+/+^ males (Fig. S2).

### TLR2^−/−^ male mice are highly sensitive to light damage

**Figure 3:**
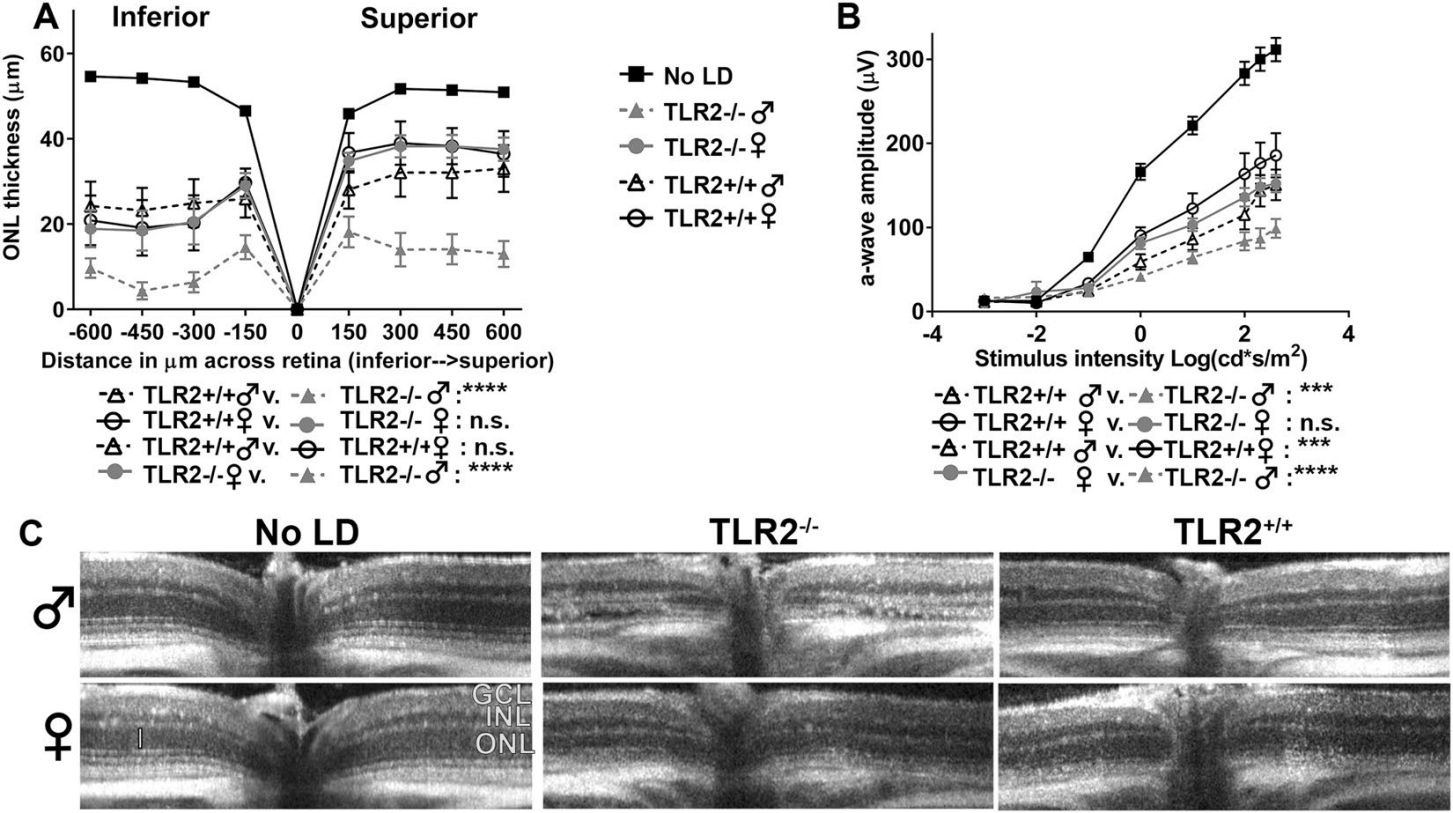
TLR2^−/−^ mice have increased susceptibility to light damage (LD). (**A**) Thickness of the photoreceptor layer (ONL) in TLR2^−/−^ and TLR2^+/+^ mice are plotted versus the distance from the optic nerve head (0 point). Thicknesses from male and female mice are plotted separately for each genotype. Measurements were made following LD. Plots from mice not subjected to light damage (No LD) are shown for comparison. (**B**) Scotopic ERG a-wave amplitudes are plotted versus intensity of light stimulation. Analysis of males and females are shown separately following light damage. (**C**) Representative OCT b-scans that were used to quantify retinal thickness shown in A. P values are from two-way ANOVA with a Sidak correction for multiple comparisons. n = 8–13 total from 3 independent experiments.

LIF and Hmox1 are known to contribute to photoreceptor survival in light stress^2,30^. As TLR2^−/−^ mice exhibited a blunted induction of LIF, Hmox1 and other stress-inducible genes in LD, we hypothesized that they would also be more sensitive to LD. To test this, we exposed TLR2^−/−^ and TLR2^+/+^ control mice to light damage and examined retinal structure using OCT and function using ERG one week later. Light exposure caused a reduction in ONL thickness, most notably in the inferior retina (Fig. 3A), and reduced scotopic a-wave amplitudes relative to untreated mice (No LD) (Fig. 3B). For simplicity, the No LD group includes all mice in the absence of light damage. As mentioned above, there were no sex-dependent differences in ONL thickness or ERG a-wave amplitudes in the absence of light damage (Fig. S1). We found that male mice of either genotype had a greater reduction in ERG a-wave amplitudes than their female counterparts, but this difference was much more pronounced in TLR2^−/−^ male mice. When compared to TLR2^+/+^ male mice, TLR2^−/−^ male mice had a greater reduction in ONL thickness (Fig. 3A), and a greater reduction in scotopic a-wave amplitudes (Fig. 3B). Additionally, male TLR2^−/−^ mice exhibited a greater reduction in ONL thickness than female TLR2^−/−^ mice with light damage. Representative OCT images are shown in Fig. 3C. Consistent with increased sensitivity to light damage, TLR2^−/−^ male mice showed an increase in hyper-reflective material in the ONL during the course of degeneration following light damage (Fig. S3). This observation was evident beginning at 8 hr following LD and persisted an extended period of time in TLR2^−/−^ male mice.

### Activation of TLR2 protects mice from light damage and activates STAT3

In Fig. 2 we showed that in the absence of TLR2, induction of protective stress responses is blunted, particularly when comparing male mice, and in Fig. 3 we showed that in the absence of TLR2, males are more sensitive to light damage. We reasoned that if TLR2 is protective, then stimulation of TLR2 should lead to protection and cause induction of protective pathways. To test this idea, we injected BALB/cJ mice intravitreally with a known ligand to TLR2 (Pam3CSK4) or vehicle (PBS). Following injection, retinas were collected for quantification of pSTAT3 using western blot. pSTAT3 levels were low 1 hour following injection with either PBS or Pam3CSK4, but were elevated at four hours following injection. Intravitreal injection requires puncturing the eye, and is known to cause retinal STAT3 activation in vehicle controls^31^. Injection of Pam3CSK4, led to much higher pSTAT3 levels than PBS injection four hours following injection (Fig. S4A and S4B). As stimulation of TLR2 led to phosphorylation of STAT3, we reasoned that Pam3CSK4 injection should also protect mice from light damage. We hypothesized that LIF-mediated STAT3 activation was responsible for protection in mice injected with Pam3. To test this idea, we injected retina-specific Gp130 knockout mice (Gp130 rKO mice) and controls with Pam3 followed by light damage. We first compared Cre- control mice injected with Pam3 and found that Pam3 injection led to preservation of ONL thickness following light damage in both males and females (Fig. S4C). Next, we asked whether Gp130 was necessary for Pam3-mediated protection in both males and females (Fig. S4D and S4E) by comparing Gp130 rKO mice with controls. We found that Pam3-mediated protection was only partially dependent on Gp130, and that female Gp130 rKO mice, but not male Gp130 rKO mice were significantly protected from light damage by Pam3 (Two-way ANOVA). Coupled with our observations in Fig. 3, these data show that TLR2 activation can induce protection in both males and females, but is essential for photoreceptor survival only in males, and that Gp130 plays a partial role in TLR2-mediated protection from light damage.

### TLR2 and other PRRs are highly enriched in resting microglia and inducible in Müller cells

We show that TLR2 is involved in the induction of protective stress responses, but it is not known which retinal cell type is responsible for TLR2-mediated protection of photoreceptors. TLR2 is known to be expressed in microglia and macrophages^28,32^. To determine whether other cells in the retina express TLR2 we analyzed publicly available data of flow-sorted cells. In Siegert *et al*.^32^, more than 20 different mouse strains were used to express GFP under cell-specific promoters in the retina, including more than 20 neuronal and glial retinal cell types. Using this strategy, microarray data were generated from many retinal cell populations under normal, unstressed conditions. TLR2 was reported to be enriched in microglia, but it was unclear to what extent, and was unclear whether other retinal cells may be involved in TLR2-mediated protection^32^. We examined this dataset to determine to what extent TLR2 and related signaling is enriched in microglia relative to other retinal cells (Fig. S5). As expected TLR2 was highly enriched in microglia and expressed at much lower levels in ganglion cells, and essentially undetectable in other cell populations. For genes in Fig. S5, the expression level in microglia was divided by the expression level in the next highest-expressing cell. The result is called the specificity ratio^32^. For TLR2, this was very high at 27.2. Necessary coreceptors for TLR2 signaling, TLR1 and TLR6 had specificity ratios of 18.5 and 17.5, respectively. This suggests that DAMP-induced protective stress responses in LD depend on microglial TLR2.

One disadvantage of this dataset was that Müller cells, another candidate cell for DAMP recognition were not included. To investigate whether Müller cells express TLR2, we accessed another publicly available dataset^33^. These data are from microarray analysis of single Müller cells from wild type (WT) mice or mice with inherited retinal degeneration (Rho^−/−^, Rhod KO mice)^33^. In this dataset, TLR2 expression was generally very low in untreated WT Müller glia. In Rho^−/−^ mice at the peak of rod degeneration (8 weeks), TLR2 expression was consistently upregulated in Müller cells, and was reduced once all rods were absent at 25 weeks of age. Interestingly, and consistent with our findings, LIF and Hmox1 induction followed a similar pattern as TLR2 induction (Fig. S6(i), top). Next, we examined the expression of other pattern recognition receptors (PRRs), many of which were also induced in Müller cells (Fig. S6A(i)). The induction of PRRs was selective since other PRRs examined were not induced (Fig. S6Aii). To identify transcriptional programs that might regulate these PRRs, we analyzed the data for transcription factors normally expressed in microglia and found that they were also upregulated in 8 week-old Rho^−/−^ mice (Fig. S6B(i)). Of these candidate transcription factors, IRF8 was one of the most highly expressed and consistently upregulated. IRF8 has been shown to be involved in the induction of TLR2 *in vitro* and *in vivo*^34^. It is possible that IRF8 may regulate TLR2 and other PRRs in Müller cells. From these data, we infer that TLR2 expression is inducible in Müller cells subsequent to damage, but is highly expressed in untreated microglia (Figs S5 and S6). These two observations suggest that microglia may be involved early in recognizing DAMPs via TLR2 following an acute insult such as LD, while upregulation of DAMP receptors in Müller cells may occur in a more long-term stress, such as that caused by an inherited mutation.

### Microglia phenotype following light damage depends on TLR2

**Figure 4:**
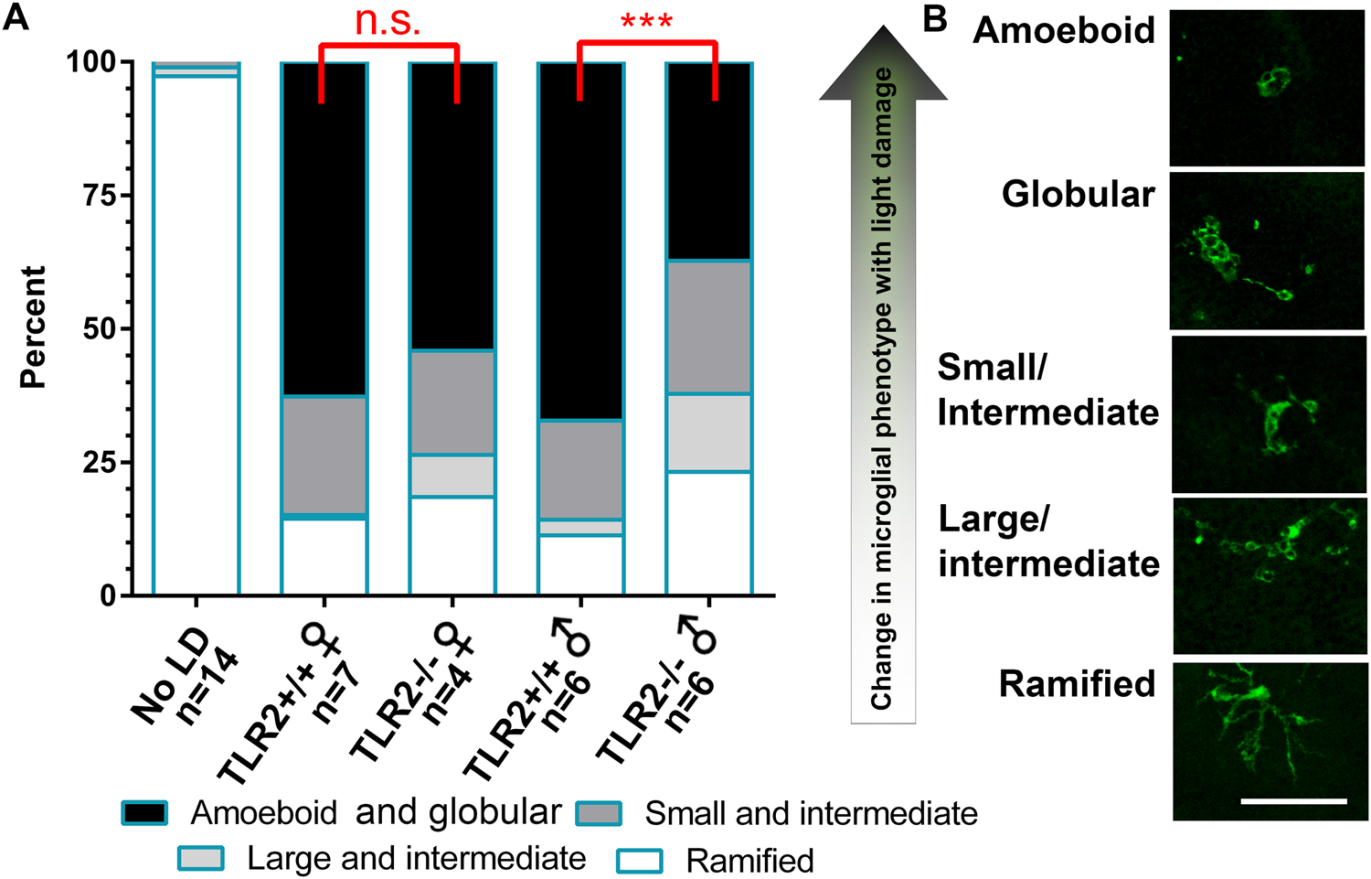
Microglia phenotype following light damage. Mice were exposed to light damage (LD). Eight hours following LD, flat mounts were made as described in methods, and stained with IBA1. (**A**) The proportion of microglia different activation states was calculated and plotted. (**B**) Microglia were counted from four consistent positions adjacent to the optic nerve, and sorted into categories, depending on their shape by a masked observer. n = 14 (No LD), and n = 4–7 mice exposed to light damage, combined from two independent experiments. Number of mice in specific groups is shown in the figure. Statistics: Two-way ANOVA with SIDAK post hoc test. The scale bar shown in (**B**) is 50 µm.

As we observed that TLR2 expression is highly enriched in microglia relative to other retinal cell types and that Müller cells do not appear to express TLR2 at baseline, we hypothesized that microglial responses following light damage depend in part on TLR2. To test this possibility, we exposed TLR2^−/−^ and controls to light damage and examined microglial morphology at eight hours post light damage, a time point that is just before the onset of photoreceptor death. To examine microglia phenotype, we stained flat-mounted retinas from mice exposed to light damage with anti-IBA1. In the retina, IBA1 is a marker for microglia. As expected, light damage caused microglia to adopt a more activated phenotype, characterized by rounded cell bodies, and retracted processes. A masked observer counted microglia and organized them into groups, based on shape, a stand-in for response to light damage. The proportion of microglia in each group was calculated and is shown in Fig. 4. In mice not exposed to light damage of both sexes and genotypes, the vast majority of cells are ramified and no clear differences were observed while comparing mice in the absence of light damage. A comparison of sex-specific groups in mice not exposed to light damage is provided in Fig. S9. Light damage caused a change in microglia phenotype, marked by an increase in the proportion of amoeboid and other intermediate groups, and a decrease of cells in the ramified cell group. As we consider amoeboid cells to have had the strongest response to light damage, we asked whether TLR2^−/−^ mice had fewer amoeboid cells following light damage than TLR2^+/+^ male mice. We found that TLR2^−/−^ male mice have a smaller proportion of activated microglia including those with amoeboid and globular morphology (p < 0.05, Two-way ANOVA). However, the differences were not statistically significant comparing female mice. The reduction in activated microglia in male mice was accompanied by an increase in ramified cells, but the differences did not reach statistical significance. These data suggest that TLR2 is required for normal microglial activation following light damage, in a sex-dependent manner.

## Discussion

In previous studies, we and others have shown that, early in retinal degeneration, LIF expression in Müller cells slows the rate of retinal degeneration by acting on rod photoreceptors^3,5,29,33,35^. The purpose of this study was to identify mechanisms that mediate stress-inducible protective responses. We determined that retinal TLR2 is necessary for induction of protective stress response genes, including LIF and Hmox1. Using publically available data, we show that TLR2 and related signaling is enriched in microglia with no treatment. Interestingly, TLR2 and other PRRs were also found to be inducible in Müller cells early in the course of progressive retinal degeneration, suggesting that Müller cells may become able to respond to stress directly during the course of a more chronic neurodegenerative condition. The results seem to be specific to TLR2 since pilot studies using TLR4^−/−^ mice had normal induction of protective response and were not more sensitive to light damage. Therefore we focused on the role of TLR2 rather than the role of TLR4.

An interesting and pervasive finding in our study was that male mice have a greater dependence on TLR2 than female mice. This was observed in induction of stress response genes, including LIF and Hmox1 (Fig. 2), photoreceptor survival (Fig. 3) and even microglial response to light damage (Fig. 4). Previously published work has shown that LIF^−/−^ mice are sensitive to LD^2^, resembling the phenotype of TLR2^−/−^ mice. Induction of stress-inducible genes and LIF was highest in TLR2^+/+^ male mice. LIF was decreased in both TLR2^−/−^ males and females relative to their TLR2^+/+^ counterparts, though this observation was only statistically significant when comparing male mice. These data suggest that male mice generally respond more strongly to LD than females, and that protective stress responses in male mice are dependent on TLR2. We additionally found that TLR2 activation-mediated protection by Pam3CSK4 is partially dependent on Gp130 in males, but not in females (Fig. S4). Our results suggest that female mice may have additional mechanisms that protect photoreceptors from light damage. There are a number of other studies that describe sex-dependent phenotypes in TLR2^−/−^ mice^36–39^. However, many studies involving TLR2^−/−^ mice have only included males, which leads to a reduction in available information^19,21,22,27^. The reason for a sex-specific phenotype in TLR2^−/−^ mice is not entirely clear but may be worthy of future investigation. Recent studies have shown that estrogen and tamoxifen protect mice from light damage, making sex hormones good candidates for alternative protective pathways^40,41^. Interestingly, and of relevance to this work, estrogen signaling has been shown to influence microglial activity^42–44^. This may partially explain why TLR2^−/−^ male mice specifically, have reduced number of amoeboid cells relative to their TLR2^+/+^ counterparts. Observations of sexual dimorphism in neurodegenerative disease is not uncommon, and sex hormones have well-established roles in neuroprotection. There are clear and well-known sexual dimorphisms in age-related macular degeneration, alzheimer’s disease, parkinson’s and multiple sclerosis, among others^45–48^.

While TLR2 was not found to be necessary for photoreceptor survival in females, TLR2 activation can still protect them from light damage (Fig. S4). As a whole, our experiments strongly suggest a role for TLR2 in the induction of protective stress responses. TLR2 activation led to delayed STAT3 activation at four hours post-injection, which suggests the activity of newly transcribed genes, likely including LIF. Our data also shows that TLR2 activation protects mice from light damage in a manner that is partially dependent on Gp130 (LIF receptor, Fig. S4). This leaves the possibility that TLR2 activation leads to multiple protective pathways, including LIF and Hmox1. This hypothesis makes sense, as in stress or neurodegeneration, the recognition of DAMPs occurs at an early stage.

DAMP-dependent induction of stress responses likely occurs primarily in glial cells. TLR2 is highly enriched in resting microglia, but is consistently induced in Müller cells under stress (Figs S5 and S6). We chose to measure gene expression at 4 hours after the onset of LD, a time point that is not confounded by rod photoreceptor death. This time point is also likely too early for Müller cells to both induce TLR2 and subsequently induce gene expression changes downstream of TLR2. As LIF is known to be protective and expressed in Müller cells, microglia are likely acting as an intermediate in the induction of Müller cell protective responses. Indeed, it has been shown that microglial mobility depends in part on TLR2^49^. One explanation to our data showing that TLR2^−/−^ male mice have reduced formation of amoeboid cells, is that microglia have an impaired ability to move toward areas of stress (i.e. photoreceptors) in response to stimulation with DAMPs. One study has shown that microglia-Müller cell interactions lead to changes in Müller cell phenotype, as well as induction of LIF and other neurotrophic factors^35^. This paradigm makes sense, as our data show that TLR2^−/−^ mice have a large attenuation in LIF induction, but not a complete loss of induction. Accordingly, it seems likely that there are multiple signals which cause LIF induction. Ligands to TNF receptors are likely candidates for microglia-derived intermediates that signal to Müller cells. TNFα specifically, is known to stimulate LIF expression in the retina^50^. TNFα is known to be induced in microglia, but not astrocytes, in a TLR2-dependent, but not TLR4-dependent manner^51^. Additionally, microglia-expressed genes IL-1β and TNFα were found to be expressed in primary microglia following stimulation with photoreceptor proteins^24^. However, we measured TNFα in our studies, but did not observe a reduction in TNFα expression in TLR2^−/−^ mice immediately after light damage (data not shown). Thus, there is likely another microglia-derived intermediate which leads to the induction of LIF expression in Müller cells.

**Figure 5:**
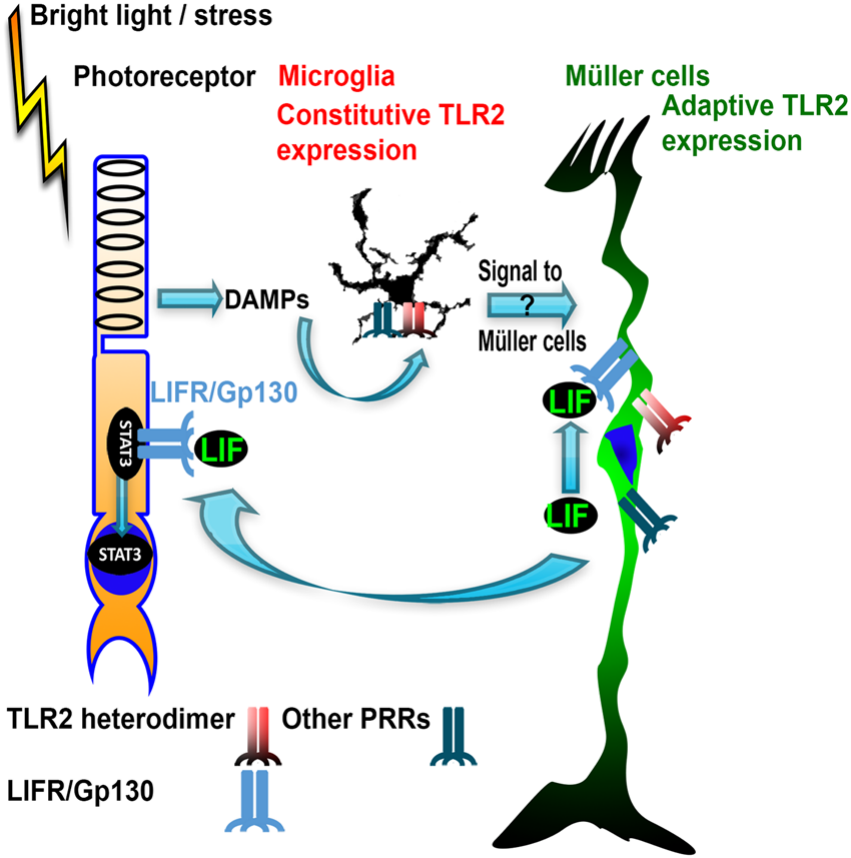
Proposed model of stress-induced LIF expression. This model is constructed from our data and previous data published in the literature from our lab and others. Stress, either from acute light damage or inherited mutation, induces the formation of DAMPs, which signal to TLR2 and other receptors on microglia. Microglia then produce an intermediate signal to induce Müller cell stress responses, including LIF expression to protect photoreceptors and slow degeneration. In addition, Müller cells upregulate TLR2 and other PRRs allowing them to directly respond to DAMPs.

The induction of PRRs in Müller cells suggests that, over time, macroglia can enter a state where they can respond to a wider range of DAMPs directly, which may be beneficial in degenerating retinas. Our analysis identified a group of transcription factors which may be responsible for PRR induction and correlate with LIF and TLR2. It is interesting to speculate that Müller cells stress responses include a transcriptional network that is responsible for upregulating PRRs and protective genes. Part of this state may include the upregulation of IRF8, a transcription factor that has been mainly studied in microglia and macrophages, and that we have now identified in Müller cells. IRF8 has been shown to be involved in induction of TLR2 expression in spinal cord following peripheral nerve injury^34^. Figure 5 shows our overall model, and current hypotheses for DAMP recognition and LIF induction through TLR2, which was constructed taking into account data from this paper and previous publications.

In this study, we found a beneficial role for TLR2 as a DAMP receptor that can initiate protective responses. TLR2 has also been found to be beneficial to nervous system pathologies in the retina and elsewhere^28,52,53^. However, TLRs and TLR2 have been found to play roles in disease pathogenesis. It is not uncommon to observe alternate roles for TLRs, depending on disease model or the magnitude of stimulation. Under low level stimulation by DAMP production in a slow degeneration model, the stimulation of TLR2 may initiate protection. In contrast, during a high-level stimulation, as would occur following bacterial infection, TLR2 might also participate in damaging inflammatory responses. Another critical factor that is often overlooked is the identity of the ligand, as receptors can have alternate downstream signaling dependent on the ligand. A recent paper was published which supports this idea, showing that one specific DAMP, carboxyethylpyrrole (CEP), has no effect in inducing inflammatory cytokines in primary RPE cells when used alone, but can induce inflammatory responses when co-stimulating with Pam2CSK4, a known exogenous bacterial TLR2 ligand^54^. Studies have shown that TLR2 plays a role in pathological vessel formation by CEP, an endogenous TLR2 ligand, which is highly relevant to retinal degeneration^15,17,54^. However, data are conflicting on the role of CEP in neovascularization in the eye^55^. In this study, we observed no obvious changes in retinal vasculature. During inherited retinal degeneration, retinal neovascularization is not a typical complication, in fact vessels are actually attenuated in retinitis pigmentosa patients^56^.

In 2005 and 2006, Rattner *et al*. presented data and described an interesting hypothesis where induction of protective gene expression in the retina likely evolved to protect the retina from retinal detachments or tears, extensive exposure to sunlight, or infection^8,29^. Our results support this observation and extend it by describing an upstream role for the PRR, TLR2 in the induction of critical neuroprotective pathways in the retina. Given the preponderance of data showing that induced expression of LIF can delay retinal degenerations, this study now links that activation of PRRs to this induced protective response^1–4,57^. Our data suggests that TLR2 activation leads to additional protective pathways. An attractive perspective is that the nervous system adapted signaling from the innate immune system to develop a coordinated protective response to a broad range of photoreceptor injuries. Further defining these pathways may lead to additional generally applicable neuroprotective therapies.

## Materials and Methods

### Mice

All mice were treated in accordance with the ARVO Statement for the Use of Animals in Ophthalmic and Vision Research, and were approved the IACUC at the University of Florida. TLR2^−/−^ mice in this paper were B6.129S1-TLR2^tm1Dgen^/J that were put on the BALB/cJ background and bred for 6 generations before generating congenic controls and knockout mice from heterozygous parents. During backcross to BALB/cJ and subsequent breeding, mice were screened for removal of the rd8 mutation and the RPE65^Met450^ mutation TLR2 transcripts were not detectable by qRT-PCR in TLR2^−/−^ mice (Fig. 1E). For all experiments, a concise table of the number of mice used in sex-specific groups can be found in Tables S1 and S2.

### Light damage

Mice at 8–14 weeks of age were placed in darkness for at least 8 hr prior to light damage. Light damage was performed in normal mouse cages (Allentown) with modified cage lids containing strips of LEDs emitting 5500 K light. Light damage was performed in a ventilated rack with automatic watering. Damaging light at 1200 lux was delivered from 6:00 p.m. to 10:00 p.m. Power settings were calibrated using a Traceable 3251 Dual-Range Light Meter (Extech Instruments, Waltham, MA,). For *in vivo* assays, mice were moved back to dim lighting following light damage to recover prior to ERG or OCT. For Fig. 3, ONL thickness and scotopic ERGs were measured 7 days following light damage. For gene expression experiments, tissue was collected immediately following light exposure.

### Scotopic Electroretinography (ERG)

Mice were dark-adapted for 4–5 hours prior to scotopic ERGs. All ERGs were done starting at 1PM. Eyes were dilated using eye drops containing tropicamide and phenylephrine. Following dilation, mice were anesthetized using a mixture of ketamine and xylazine (100 mg/kg ketamine and 5.2 mg/kg xylazine). Lubricating eye drops were placed in each eye to stabilize corneal hydration and to serve as a conducting solution. Gold wire loop electrodes were placed on the cornea in contact with the conducting solution. A stainless steel reference electrode was placed in the cheek, and a ground needle electrode was placed in the tail. Mice were exposed to an increasing series of light stimulations using an Espion ColorDome™ (Diagnosys LLC, Lowell MA, USA), and a xenon light source was used for stimulation. ERG a-wave amplitudes were measured, transferred to Excel and plotted versus stimulus intensity. All ERGs performed on light damaged mice were done one week following light damage. All ERGs were don in the afternoon, starting at 1PM.

### Spectral Domain Optical Coherence Tomography (OCT) and Outer Nuclear Layer (ONL) measurements

Mice were anesthetized following the procedure described for scotopic ERGs. Each mouse was placed on a movable pedestal for retina visualization by OCT, and lubricating eye drops were placed in each eye to stabilize corneal hydration. Just prior to imaging, excess drops were removed using cellulose spears (Beaver Visitec, Waltham, MA). The mouse was oriented in the holder to consistently place the optic nerve in the center of the imaging area. Rectangular volume scans of the retina were obtained using OCT system (Envisu R2200, #90-R2200-V1–120). Volume scans were analyzed for retinal layer thickness by auto-segmentation and ONL thickness by manual calipers using Bioptigen Diver software (Leica, Wetzlar, Germany). Manual ONL thickness measurements were done using nine evenly spaced points from the superior to inferior retina, centered on the optic nerve. Unless otherwise mentioned, all OCTs and ERGs performed on light damaged mice were done one week following light damage.

### Western Blot

Retinas were dissected in ice cold, sterile PBS containing protease inhibitors (#20-201, Millipore, Billerica, CA) and phosphatase inhibitors (#P0758S New England Biolabs, Ipswich, MA). To preserve protein following collection, retinas were flash frozen in liquid nitrogen and stored at −80 °C until further use. Proteins were prepared by sonicating retinas, and clarification by centrifugation at 12,000 × g. Protein concentrations were measured using the BCA assay (ThermoFisher, Waltham, MA), and western blots were done as previously described^31^. Antibodies used: pSTAT3 Y705 (1:2,000 Rabbit, #9145), total STAT3 (1:2,000, Rabbit, #9132 L) (Cell Signaling Technology, Danvars, MA), and actin (Goat, ab6276, Abcam, Cambridge, UK). The antibodies for phosphorylated and total STAT3 have been extensively characterized^3,4,58^. Signal detection was done using fluorescently-labeled secondary antibodies to rabbit (Goat, IRDye 800 CW) and mouse IgGs (Goat, IRDye 680RD) (LICOR, Lincoln, NE). Fluorescent signals were measured using a LICOR Odyssey imager and Odyssey Image Studio software (version 5.2). Western blots in Fig. 2 were cropped. Full, uncropped versions of these blots can be found in Figs S7 and S8.

### Quantitative real time PCR (qRT-PCR)

Retinas were dissected in ice cold, sterile PBS and flash frozen in TRIzol using liquid nitrogen. RNA was extracted using TRIzol according to manufacturer’s instructions (Invitrogen Carlsbad CA, USA). Primers were designed to amplify across a large intron, or to bind to an exon-exon junction, to avoid any potential DNA contamination. cDNA was synthesized using iScript according to the manufacturer’s instructions (Bio-Rad, Hercules, CA, USA). qRT-PCR was done using SSOfast supermix according to the manufacturer’s instructions (Bio-Rad, Hercules, CA, USA). Data were analyzed using a standard ΔΔct calculation. Data were first normalized to RPL19, followed by normalization to control mice (TLR2^+/+^ males with no LD) to get fold mRNA level relative to control mice. PCR primers are shown in Table S3. If an outlier was suspected, data were subjected to Grubb’s test, and the outlier was removed if statistically significant. Primers were validated by measuring amplification efficiency using a 2-fold dilution series. Primers were considered valid if amplification efficiencies ranged between 80 to 120%, and melt curves resolved in a single peak. Most primer pairs fell within 90–110% amplification efficiency. This information is included in Table S3.

### Intravitreal injections

Mice were anesthetized as described above. Following anesthesia, a 30 G needle was used to puncture the eye near the limbus. A second, 33 G needle was inserted into the hole created by the first needle, ensuring no damage was caused to the eye or lens. When the needle is clearly observed under the lens and in the vitreous space, the plunger was depressed slowly over a period of 20–30 seconds. Injected reagents include either 1 µL of PBS (vehicle) or 1 µg/µL Pam3CSK4 (Sigma, St. Louis, MO) dissolved in PBS. Mice were returned to cleaned cages following injection. Eyes were removed from experiments if significant injection damage was observed, including hemorrhage or significant detachment, which was uncommon.

### Histology (for photoreceptor nuclei counting)

One week following light damage, eyes were fixed in perfix, and processed in a tissue processor, followed by embedding in paraffin and H&E histology. The number of nuclei per photoreceptor column was counted at 20x magnification and plotted as a function of distance from the optic nerve.

### Data analysis from previously published microarray studies

Data were accessed from the Gene Expression Omnibus (GEO)^29,32,33,59^ Accession numbers: GSE35386, and GSE33085. Specific datasets were accessed and sorted using the Bioconductor and GEOquery packages in R. For supplementary. For Fig. S5, the specificity ratio was defined as previously described^32^. In our case, for a given gene it was the ratio of the average microglial expression to the average of the second highest-expressing group of cells. Data for heatmaps were displayed as log_2_ (fold change relative to the average of all samples). For Fig. S6, heatmaps were constructed using the pheatmap package in R. Legends were drawn to both fold induction and average expression values for each gene in Rhodopsin^−/−^ mice at 8 weeks of age.

### Retinal flat mounts

Eyes were placed in 1%PFA for 1 hour prior to dissection. The cornea was removed and the retina was separated from the RPE carefully. The lens was removed, and the vitreal membrane was carefully and completely removed. Resulting retinas were washed in 0.5% triton X-100 twice, for 15 minutes each. Following, retinas were incubated with 10% horse serum, with antibody directed against unconjugated primary antibody IBA1 (goat, Abcam) at 1:800 dilution for 6 days at 4 degrees C. Retinas were washed with agitation in 0.5% Triton X-100 twice for 30 minutes each. Retinas were placed in 10% horse serum with donkey anti-goat secondary antibodies (1:500, invitrogen) overnight. Again, retinas were washed with agitation in 0.5% Triton X-100 twice for 30 minutes each. Retinas were placed in PBS for making cuts in flat mounts. 4–5 cuts were made radially starting from the outward-facing edges. Resulting retinas were mounted on slides using 60% glycerol in PBS, and imaged on a confocal microscope. Flat-mounted retinas were also done as no primary antibody controls (see Fig. S10).

### Statistical analysis

Statistical analyses were done using GraphPad Prism software. All reported p-values were corrected for any multiple comparisons. All two-way ANOVAs with multiple comparisons used Sidak’s post-test. All t-tests were unpaired, two-tailed t-tests. For concise tables of number of mice in sex-specific groups in each experiment, please see supplemental Tables S1 and S2. For qPCR data, any clear outliers were subjected to Grubbs’ test and were removed if they were found to be statistically significant outliers. All error bars shown are SEM. Use of asterisks in figures: *p < 0.05, **p < 0.01, ***p < 0.001, ***p < 0.0001.

### Data availability

Data generated in the current study are available from the corresponding author on reasonable request. Other analyzed datasets are already publicly available on the NCBI gene expression omnibus (GEO). Accession numbers for these studies: GSE35386 and GSE33085.

## Electronic supplementary material

Supplemental material
