# Supplementary material for: Damage-associated molecular pattern recognition is required for induction of retinal neuroprotective pathways in a sex-dependent manner
Source: Sci Rep. 2018 Jun 14;8:9115. doi: 10.1038/s41598-018-27479-x (PMC6002365; doi:10.1038/s41598-018-27479-x)
Supplement: Supplementary file 1 — Supplemental material [file 41598_2018_27479_MOESM1_ESM.pdf]

Supplemental material for:

Damage-associated molecular pattern recognition is required for induction of retinal neuroprotective pathways in a sex-dependent manner

Abbreviated title: **DAMP recognition in endogenous retinal neuroprotection**

Authors:

Marcus J Hooper[1] Jiangang Wang[1], Robert Browning[1] and John D. Ash[1*]

[1]Department of Ophthalmology, University of Florida, Gainesville, FL 32610 USA

Address correspondence to:
John D. Ash PhD
Department of Ophthalmology
University of Florida
1600 SW Archer Rd
Gainesville, FL 32610

jash@ufl.edu

Table S1. Number of mice used in light damage experiments (sex-specific). The total number of mice in each sex-specific group is shown for each experiment.

	No light damage				Light damage			
	$TLR2^{+/+}$		$TLR2^{-/-}$		$TLR2^{+/+}$		$TLR2^{-/-}$	
	M	F	***M***	***F***	***M***	***F***	***M***	***F***
ERG (Figs 1, S1 and 3)	14	8	11	9	13	8	11	9
OCT (Figs 1, S1, and 3)	15	8	8	8	13	7	12	9
OCT (Fig S3)	N/A	N/A	N/A	N/A	4	3	8	7
qRT-PCR (Figs 2 and S2)	6	3	5	4	11	16	11	12
Western blot (Fig. 2)	5	0	0	0	12	4	9	8
Microglia counts (Fig. 4)	3	5	3	3	6	7	6	4

Table S2. Number of mice used in intravitreal injection experiments (sex-specific). The total number of mice in each sex-specific group is shown for each experiment involving intravitreal injections.

	Intravitreal injection (PBS left eye, Pam3 right eye)					
	BALB/cJ		Gp130f/f Cre-		Gp130 rKO	
	M	***F***	***M***	***F***	***M***	***F***
Western blot (Fig. S4)	3	0	N/A	N/A	N/A	N/A
OCT (Fig. S4)	5	0	3	3	5	3

Table S3. qPCR Primers used in this study. Primers were validated by measuring amplification efficiency using a 2-fold dilution series. Primers were considered valid if amplification efficiencies ranged between 80 to 120%, and melt curves resolved in a single peak.

Gene	Forward primer	Reverse primer	Primer efficiencies
ATF3	ACCGTCAACAACAGACCCCT	CCGCCTCCTTTTCCTCTCATC	110
CD206	GCTGGCGAGCATCAAGAGTA	AGGAAACGGGAGAACCATCAC	100
CXCL1	AACCGAAGTCATAGCCACACT	TTGTCAGAAGCCAGCGTTCA	120
GFAP	TCGACAACTGGGTACCATGC	CCTTGAAGCCAGCATTGAGC	95
Hmox1	CAGAGCCGTCTCGAGCATAG	AAGGAAGCCATCACCAGCTTA	100
IL-1b	GCCACCTTTTGACAGTGATGAG	TGCTGCGAGATTTGAAGCTG	95
INOS	TCTAGTGAAGCAAAGCCCAACA	CTCTCCACTGCCCCAGTTTT	118
IRF8	ACGCTGTGCTCTGAACAAGA	CTTCCTCGGGGACAATTCGG	90
LIF	AATGCCACCTGTGCCATACG	CAACTTGGTCTTCTCTGTCCC	101
MT1	ACCAGATCTCGGAATGGACC	CCTGGGCACATTTGGAGCAG	93
RPL19	TCACAGCCTGTACCTGAA	TCGTGCTTCCTTGGTCTTAG	96
TLR2	AAACGTGCATTCCTCAGACG	CAGCCGAGGCAAGAACAAAG	90

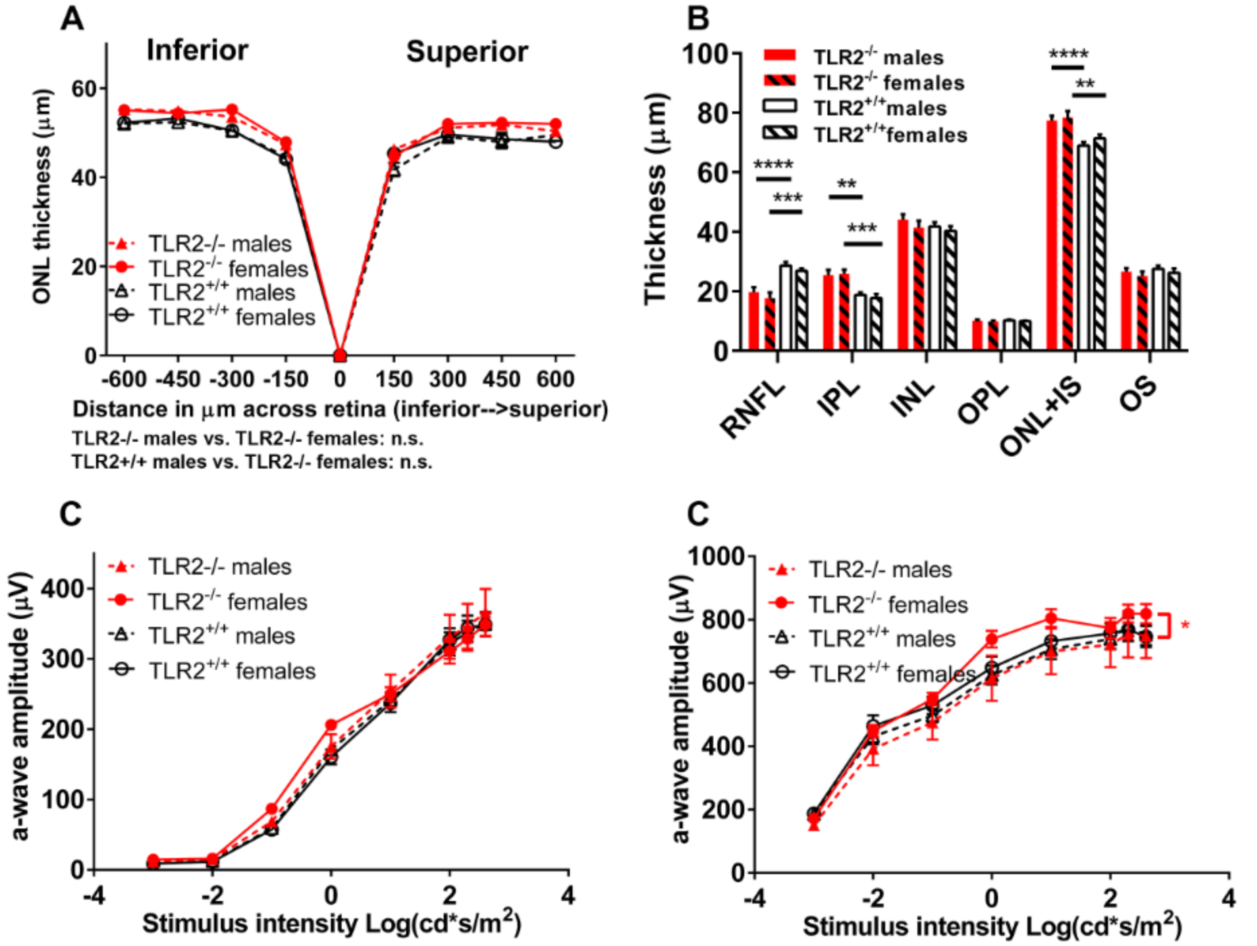

Figure S1. Retinal structure and function in 4-8 week old TLR2$^{+/+}$ and TLR2$^{-/-}$ mice (separated by sex). (A,B) Optical coherence tomography was done on TLR2$^{+/+}$ and TLR2$^{-/-}$ mice. ONL thickness was measured using calipers (A). Thickness of other retinal layers was measured using Diver software (B). (C,D) Scotopic ERG was done to measure retinal function in TLR2$^{+/+}$ and TLR2$^{-/-}$ mice. (C) a-wave amplitudes and (D) b-wave amplitudes. n=8-15 in sex-specific groups pooled from three independent experiments. For specific numbers, see Table S2. Statistics (B): Two-way ANOVA, *:p<0.05, **: p<0.01, ***:p<0.001, ****:p<0.0001.

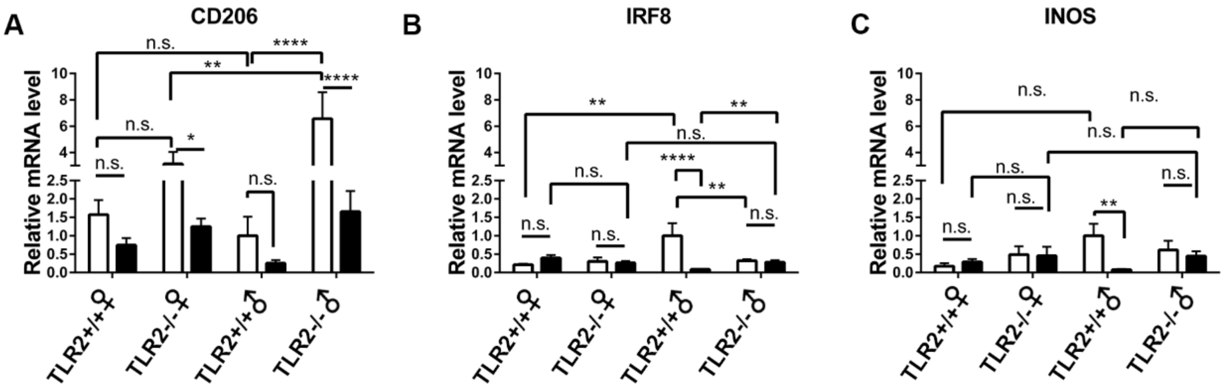

Figure S2. Microglia-expressed genes are slightly altered in TLR2$^{-/-}$ mice in the absence of light exposure. qPCR was done to measure gene expression of microglia-expressed genes **(A)** CD206, **(B)** IRF8, **(C)** INOS. The expression of these genes were not found to be dependent on genotype or gender following light damage. Interestingly, the expression of some microglia-expressed genes were different when comparing TLR2$^{-/-}$ males vs. TLR2$^{+/+}$ males. Mice exposed to light damage: n=27 TLR2$^{+/+}$ mice, 23 TLR2$^{-/-}$ mice pooled from three independent experiments. Untreated mice: n=3-6. Statistics: Two-way ANOVA. For number of mice in sex-specific groups, see Table S2. Statistics: Two-way ANOVA, *: p<0.05, **: p<0.01, ***:p<0.001, ****: p<0.0001.

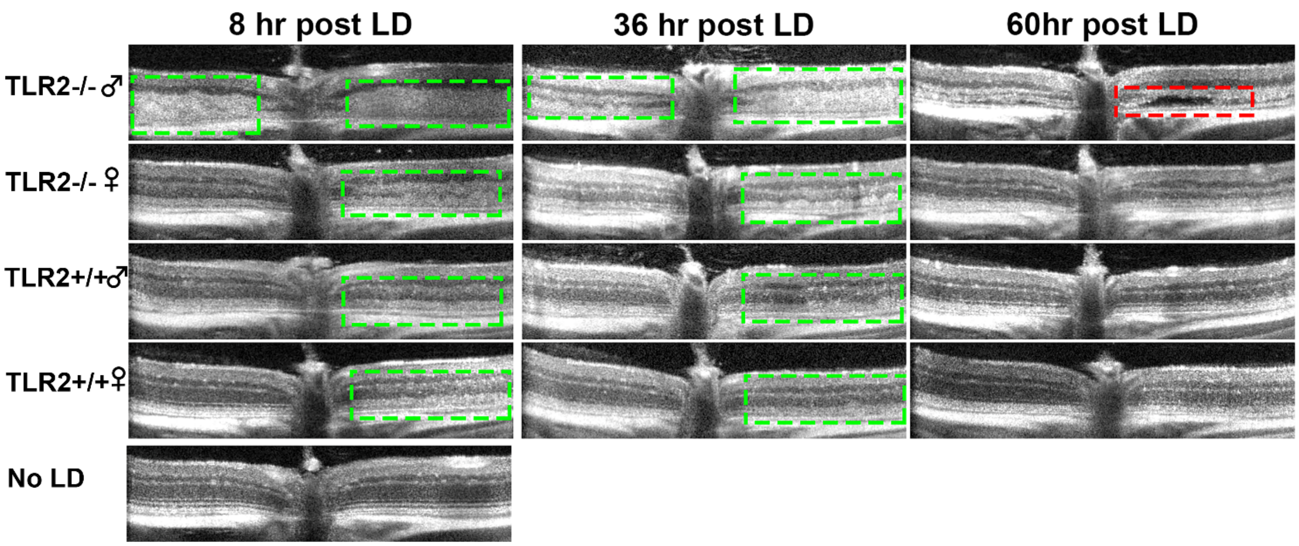

Figure S3. Male TLR2$^{-/-}$ mice have more severe and sustained structural changes in OCT reflectivity during light exposure. Mice were exposed to LD, OCT images were collected 8hrs (left), 36hrs (middle) and 60hrs (right) post LD. Shown are both males and females from TLR2$^{-/-}$ and TLR2$^{+/+}$ groups. Areas of hyper-reflection in the ONL were more prevalent in TLR2$^{-/-}$ mice and especially in TLR2$^{-/-}$ males (shown in green boxes). Hyper-reflectivity was more pronounced in males at all time points. Areas of detachment were observed frequently in TLR2$^{-/-}$ males (red box) 60hr post light exposure, but were infrequently observed in TLR2$^{+/+}$ mice or TLR2$^{-/-}$ females following light exposure. n=4-7 total from 2 experiments.

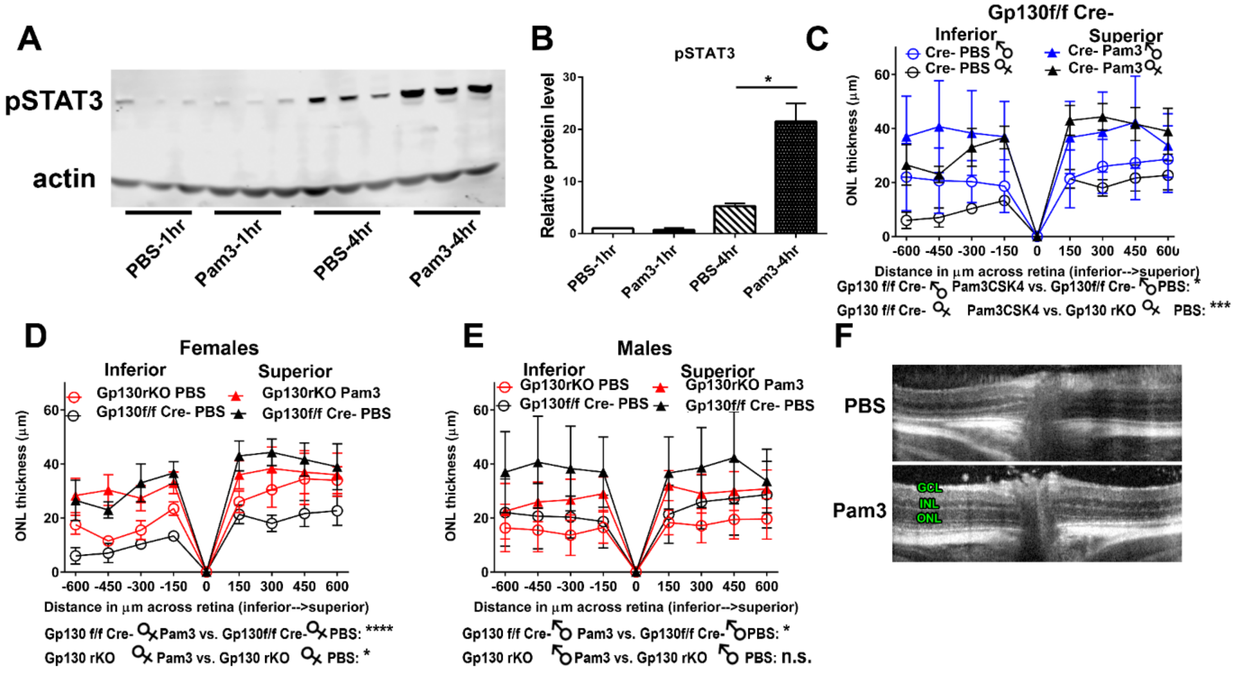

Figure S4. Activation of TLR2 activates STAT3 and protects retinas from light damage in a partially Gp130-dependent manner. BALB/cJ mice were injected with Pam3CSK4 (Pam3) or PBS as control. **(A)** pSTAT3 levels were measured at one or four hours following injection by western blot. **(B)** Quantification of data in **(A)** (n=3). **(C-F)** Gp130 retinal knockout mice and controls were injected with Pam3 or PBS (n=6-8) and exposed to light damage. (C) ONL thickness measurements in Cre- mice with the Gp130 floxed allele were injected with Pam3, followed by light damage. (D,E) ONL thickness measurements from females (D) and males (E). (F) Representative OCT images of Cre- mice injected with Pam3 or PBS. Statistics: (B) unpaired, two-tailed t-test. (C-E) Two-way ANOVA (Sidak).

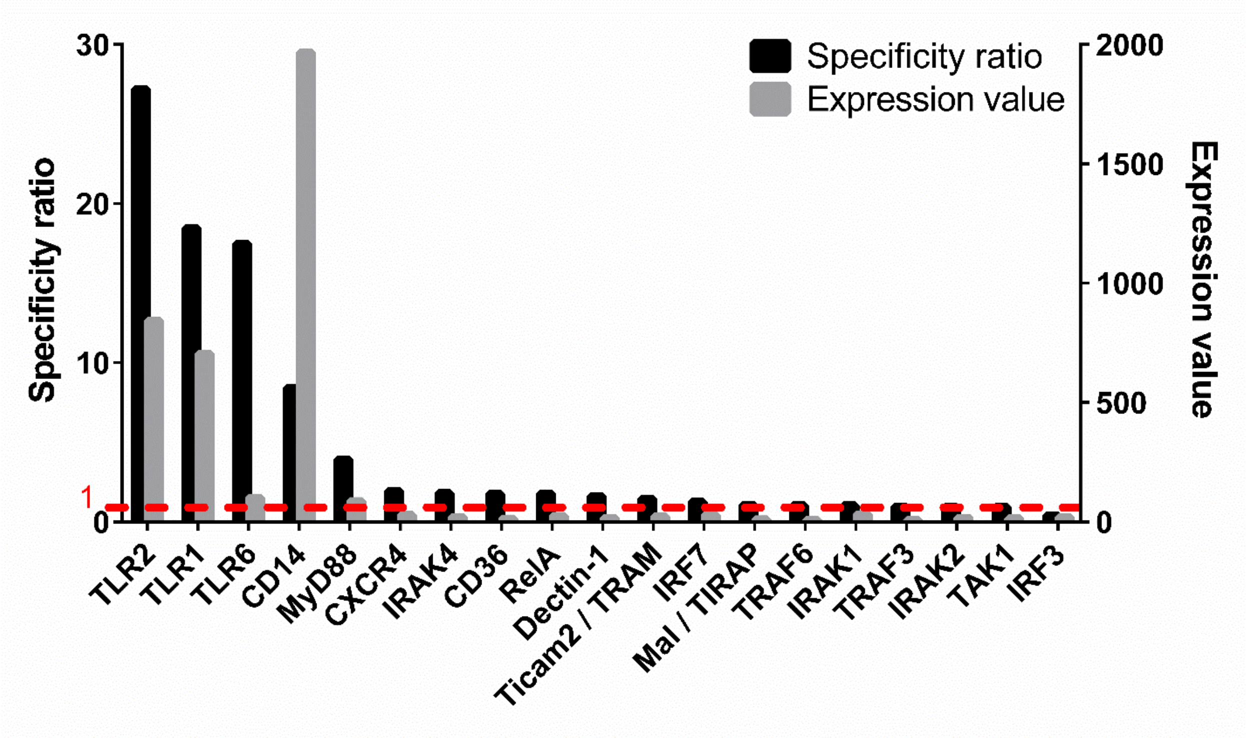

Figure S5. TLR2 and related signaling molecules are enriched in microglia. Using data from Siegert et al. 2009, we examined retinal cell type-specific expression of TLR2 and related receptors and signaling molecules. Data show DAMP receptors and genes in the TLR2 signaling pathway that are enriched in microglia**.** Specificity ratios (black bars, left y-axis, defined in methods) are used to indicate the level of enrichment of gene expression in in microglia relative to the next highest-expressing cell type. The dashed line indicates a specific ratio of 1 which is drawn to show genes that are not enriched in microglia. Gene expression values, shown in gray bars, are plotted on the right y-axis.

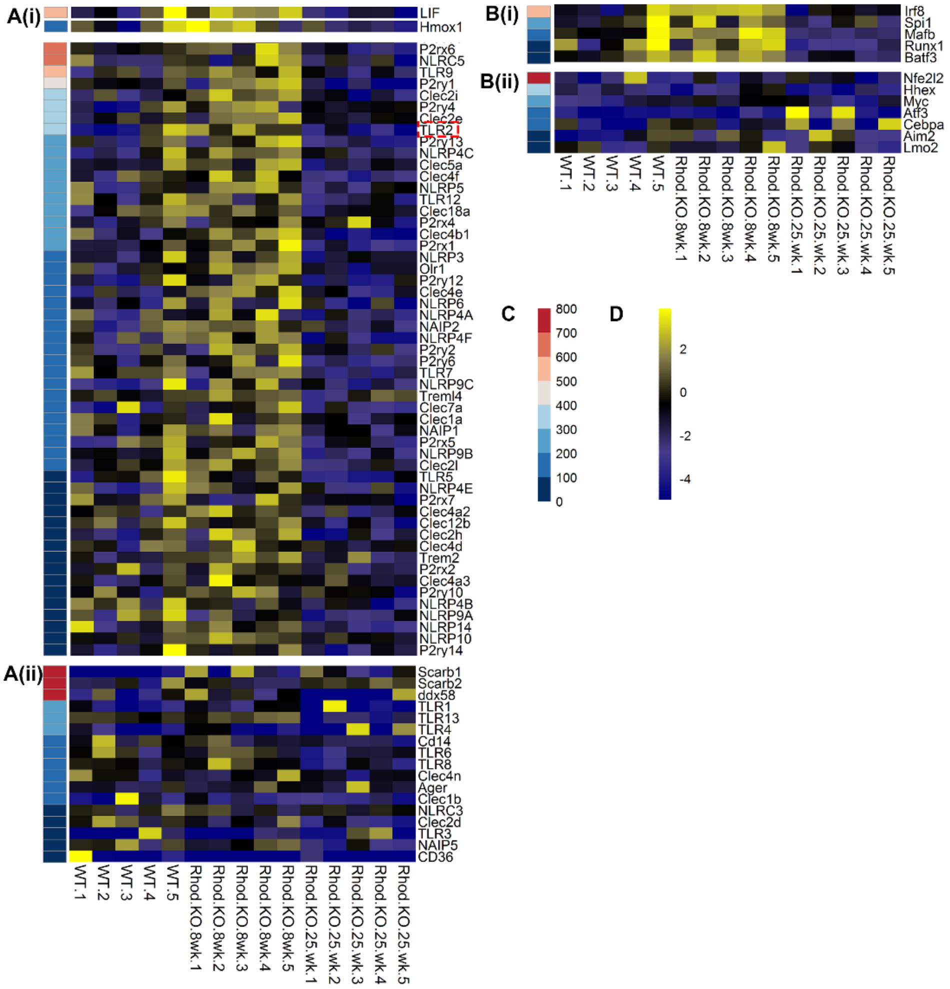

Figure S6. PRR-related gene expression induced in Müller cells at the onset of retinal degeneration. Microarray data from Roesch 2012 were reanalyzed to identify genes that are induced in Müller cells early in retinal degeneration. LIF and Hmox1 **(A(i), top)** expression are co-regulated early in the disease process. Expression of some PRRs including TLR2 (shown in red box) that have a similar induction pattern with LIF and Hmox1 are also shown **(A(i))**. However not all PRRs have the same gene induction profile and are shown for comparison **(A(ii))**. We identified other transcription factors that were also upregulated with the same profile as LIF in Müller cells from early degenerating retinas **(B(i))**. Transcription factors, which did not show this pattern, are shown for comparison **(B(ii))**. **(C)** is the intensity scale that was used to indicate the expression value for each gene shown in the left column in **A** and **B** for each gene. The color indicates the average expression level in the 8-week Müller cells from *Rho*$^{-/-}$ mice. **(D)** is the scale which indicates log2 fold change used in the heatmaps in A and B. The x-axis in **A** and **B** shows individual Müller cells.

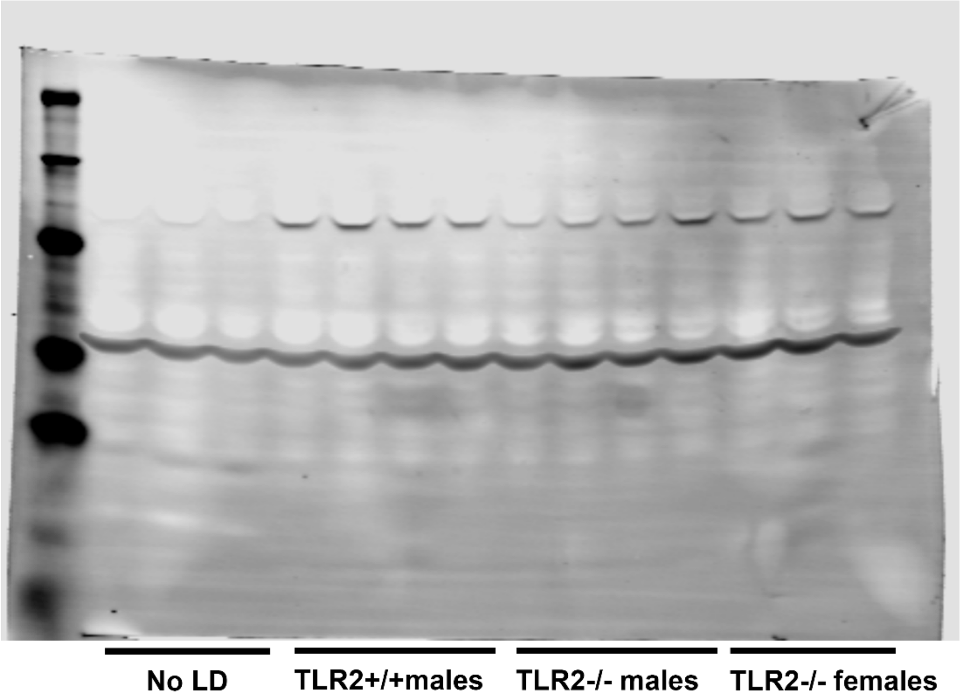

Figure S7. Full western blot for phosphorylated STAT3 from figure 2. The top band is phosphorylated STAT3, the lower band is beta actin. Mice were exposed to light damage. Immediately following light damage, eyes were collected for western blotting. n=3-4.

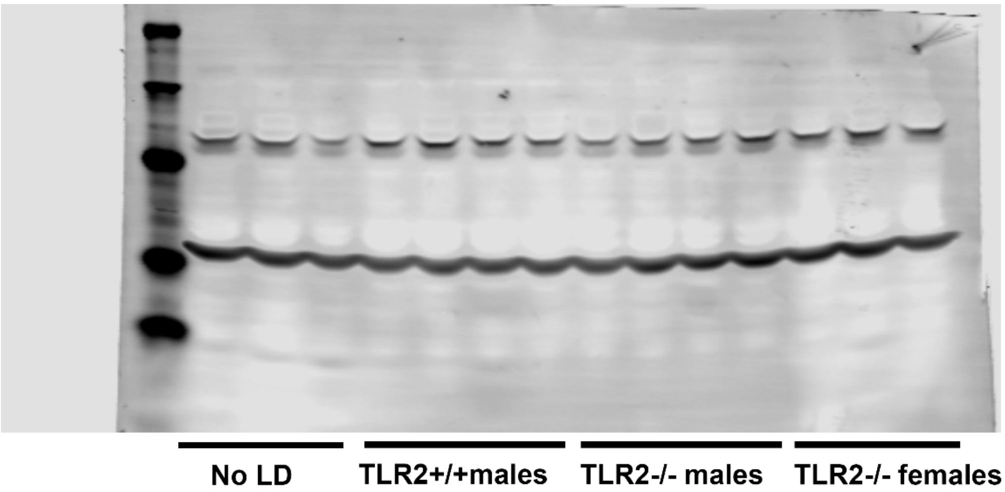

Figure S8. Full western blot for total STAT3 from figure 2. The top band is total STAT3, the lower band is beta actin. Mice were exposed to light damage. Immediately following light damage, eyes were collected for western blotting. n=3-4. This blot was obtained by probing the blot for pSTAT3 with antibodies against total STAT3.

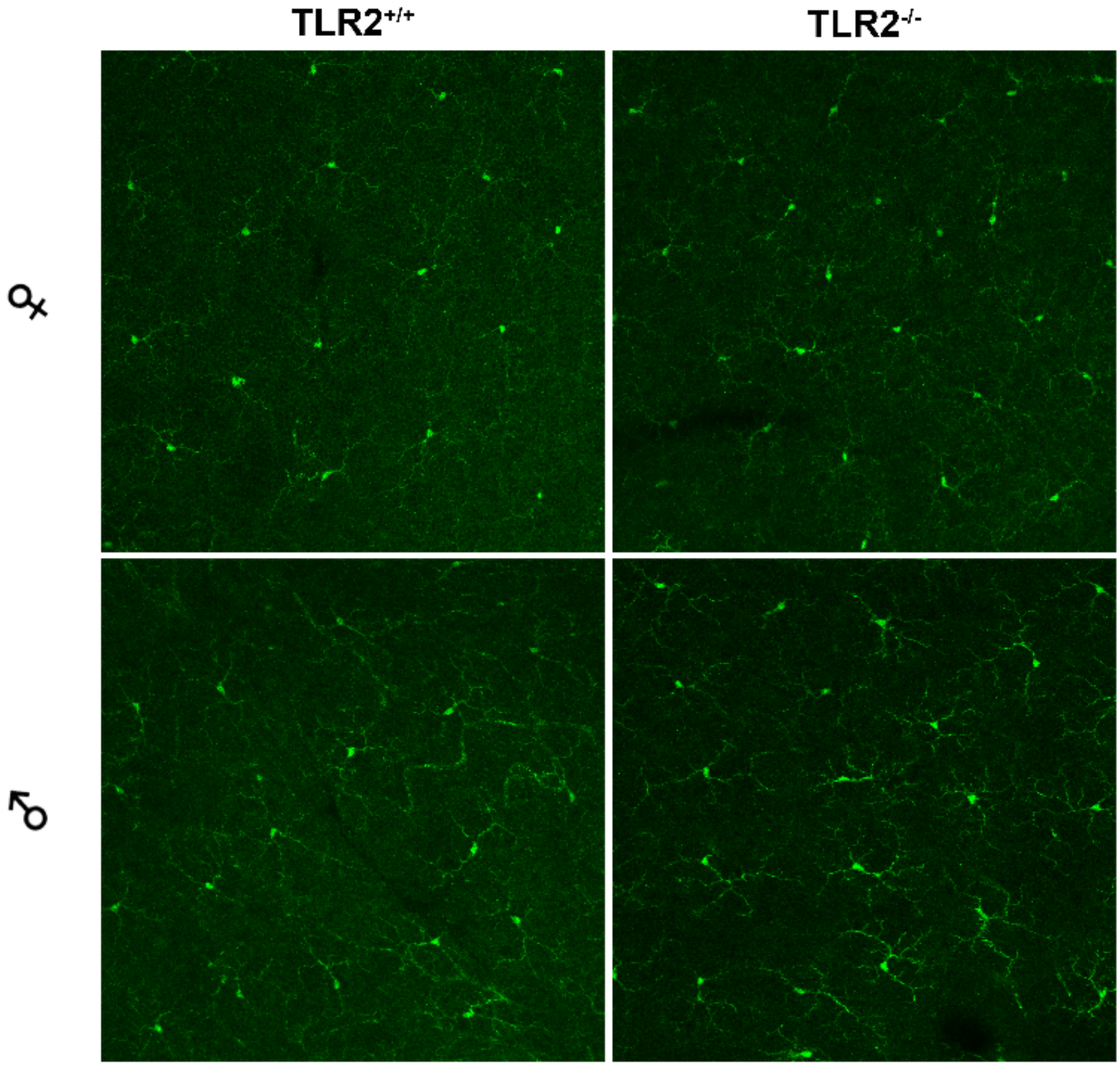

Figure S9. Comparison of untreated mice for IBA1 staining. All mice shown in this figure were not exposed to light damage. Flat mounts were made and immunostained with IBA1. The vast majority of cells in all groups were found to be ramified. n=3-5.

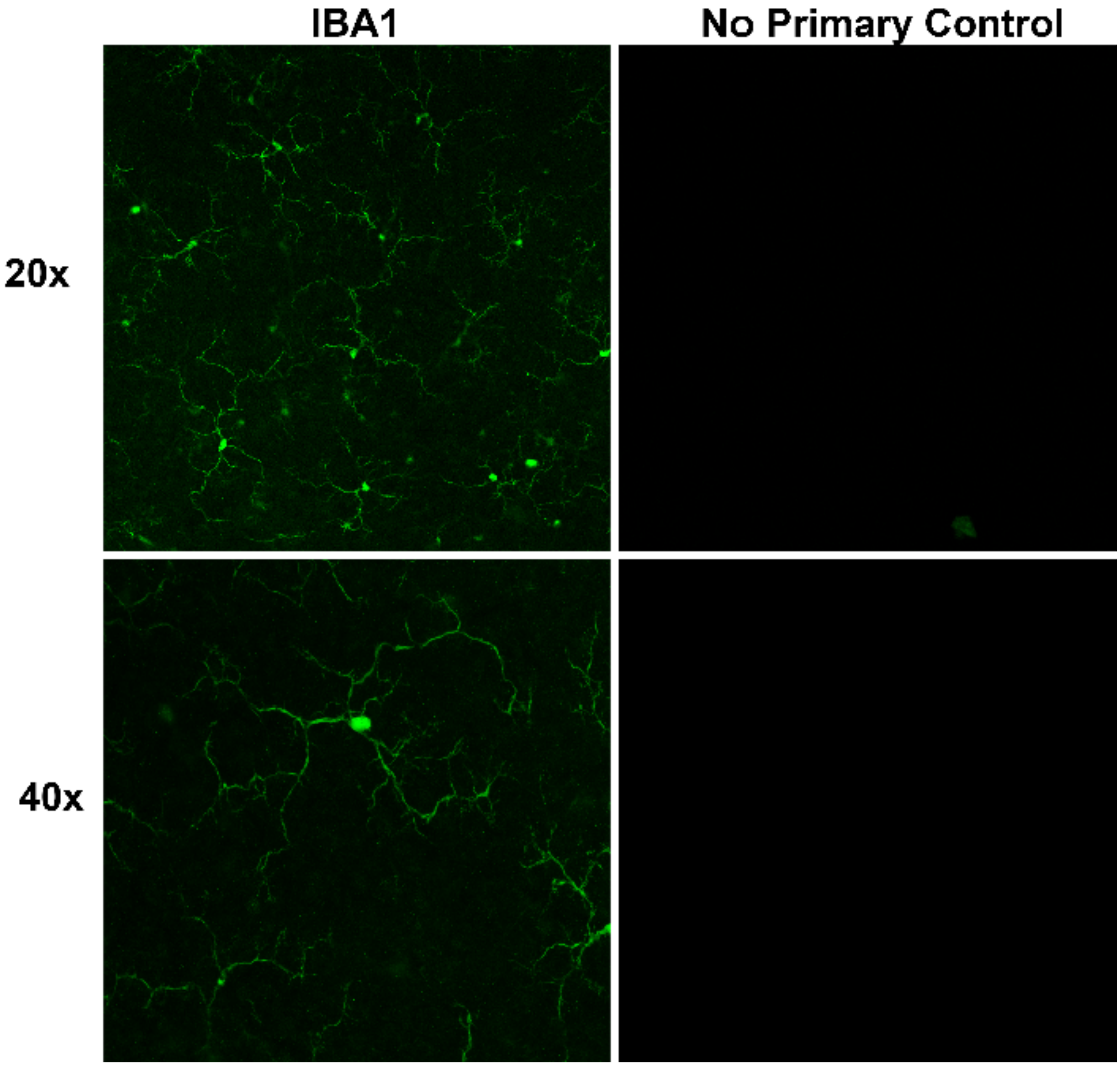

Figure S10. No primary control for IBA1 staining. Flat mounts were stained using the normal (see methods), (images on the right) or treated the same, with the omission of the primary antibody (images on the right). 20x and 40x images are shown.
